# Establishing the Safety and Efficacy of Bedaquiline-Containing Regimen for the Treatment of Drug-Resistant Tuberculosis: A Systematic Review and Meta-Analysis of Randomized Clinical Trials

**DOI:** 10.1155/2024/5542658

**Published:** 2024-08-09

**Authors:** Muhammad Candragupta Jihwaprani, Yipeng Sun, Wahyu Choirur Rizky, Idris Sula, Nazmus Saquib

**Affiliations:** ^1^ College of Medicine Sulaiman Al Rajhi University, Bukayriyah, Al-Qassim, Saudi Arabia; ^2^ Department of Anesthesiology and Intensive Care Unit RSUD Pangeran Jaya Sumitra General Hospital, Kotabaru, South Kalimantan, Indonesia

**Keywords:** bedaquiline, diarylquinolines, drug-resistant tuberculosis, meta-analysis, systematic review, tuberculosis

## Abstract

The risks and benefits of bedaquiline (BDQ) for treatment of drug-resistant tuberculosis (DR-TB) have not been firmly established. We aimed to assess the safety and efficacy of BDQ-containing regimens for the treatment of DR-TB as evidenced in available randomized controlled trials (RCTs). In this systematic review and meta-analysis, five databases (i.e., ClinicalTrials.gov, Cochrane CENTRAL, PubMed, ScienceDirect, and SinoMed) were searched. RCTs among DR-TB patients that had a control arm were eligible. The safety endpoints were all-cause mortality and serious adverse effects (SAEs). Efficacy outcomes were sputum culture conversion rate at 8–12 weeks and 24–26 weeks, treatment success, and time to culture conversion. A total of 476 records were screened; 18 met the eligibility criteria. The pooled analysis included 2520 participants (55.8% received BDQ-containing regimens, *n* = 1408). Pooled safety outcomes showed no significant reduction in all-cause mortality (relative risk [RR] [95%confidence interval (CI)] = 0.94 [0.41–2.20]) or SAEs (RR [95%CI] = 0.91 [0.67–1.23]) in the BDQ-regimen group. Pooled efficacy outcomes showed significantly superior culture conversion rates at 8–12 weeks (RR [95%CI] = 1.35 [1.10–1.65]) and 24–26 weeks (RR [95%CI] = 1.25 [1.15–1.36]), more treatment success (RR [95%CI] = 1.30 [1.17–1.44]), and a 17-day reduction in the time to culture conversion (standardized mean difference [SMD] [95%CI] = −17.46 [−34.82 to −0.11]) in the BDQ-regimen group (reference: non-BDQ regimen). Overall, BDQ regimens showed significant treatment effect against DR-TB but did not reduce mortality or SAEs.

## 1. Introduction

The burden of drug-resistant tuberculosis (DR-TB) is on the rise, reaching an estimated global prevalence of 11.6% [1]. New resistance profiles of TB have continuously emerged since rifampin-resistant TB (RR-TB) and multidrug-resistant TB (MDR-TB) were defined by the World Health Organization (WHO) in 1994 [2]. These changes in resistance profiles have rendered many TB medications ineffective, limiting the choice of effective drugs. The mortality rate is significantly higher among patients with MDR-TB (18%) and extensively drug-resistant TB (XDR-TB) (39%) compared to patients with drug-susceptible TB (DS-TB) (6%) [3].

Among the efforts to tackle this rising burden was the accelerated approval of bedaquiline (BDQ) (previously named TMC207 or diarylquinoline) for the treatment of DR-TB [4, 5]. In one of the earliest Phase 2 randomized controlled trials (RCTs), Diacon et al. reported a higher culture conversion rate and faster culture conversion rates among patients treated with BDQ compared to standard therapy [6]. Similar findings were also reported by a multicenter large-scale cohort study of 29 countries as well as more recent RCTs [7–9]. Nearly 70 countries worldwide have adopted the use of BDQ in their DR-TB therapeutic regimens [4]. BDQ was a milestone in a new era of the fight against DR-TB.

To our knowledge, four meta-analyses are available on the safety and efficacy of BDQ-containing regimens for the treatment of DR-TB [10–13]. These meta-analyses have methodological limitations. For example, they included both RCTs and observational studies. Observational studies, by design, cannot eliminate confounders and tend to have more biases; their inclusion may have increased the precision of the estimates but at the cost of inflated bias. Another limitation is that single-arm trials and trials with DS-TB patients were included in the analysis [11]. These may explain the difference in their findings; Wang, Wu, and He [10] found a significant reduction in all-cause mortality with BDQ-containing therapy while Tong et al. [11] demonstrated insignificant mortality benefits. Similarly, pooled analysis of nonrandomized studies (NRS) by Tong et al. [11] demonstrated significant treatment success, contradicting Wang et al., which found an insignificant improvement in overall treatment success [10, 11].

We have addressed these limitations and added a strength to this current systematic review and meta-analysis of BDQ by including (1) only RCT evidence, (2) RCTs with a control arm, (3) RCTs with all relevant outcomes, and (4) RCTs from China by searching a Chinese database. The objective of this meta-analysis was to determine the safety and efficacy of BDQ in the treatment of DR-TB.

## 2. Materials and Methods

### 2.1. Protocol and Registration

This systematic review and meta-analysis was conducted according to the guidelines of the Preferred Reporting Items for Systematic Reviews and Meta-Analysis (PRISMA) protocol. A comprehensive protocol was developed and submitted prospectively to the International Prospective Register of Systematic Reviews (PROSPERO) (identification number CRD42023426999).

### 2.2. Literature Search Strategy and Eligibility Criteria

In order to identify all available RCTs, five databases were systematically explored: ClinicalTrials.gov, Cochrane CENTRAL, PubMed/MEDLINE, ScienceDirect, and SinoMed. The authors searched for all available studies published through mid-December 2023. Two different search strategies were implemented: using the Medical Subject Headings (MeSH) keywords and using text field search. The following predefined keywords were used to search and identify all relevant RCTs: “bedaquiline,” “diarylquinoline,” “drug-resistant tuberculosis,” “TMC207,” and “TMC208.” The keywords were translated into Chinese by author YS, a native Chinese speaker, to find the relevant records within the SinoMed scientific database. In addition, the references of the retrieved studies were comprehensively screened to identify and retrieve any potentially relevant RCTs.

This review included RCTs that fulfilled all the following criteria: (1) participants with DR-TB (including RR-, MDR-, pre-XDR-, and/or XDR-TB) in both the intervention and control arms, (2) an intervention arm with a BDQ-containing regimen, (3) a BDQ-free control arm, and (4) inclusion of safety and/or efficacy outcomes. The retrieved records were screened independently by three reviewers (MCJ, IS, and YS) to identify duplicates and fulfillment of eligibility criteria. Any discrepancy was solved among the three reviewers and the fourth coauthor.

### 2.3. Data Extraction

Following a full-text review of the screened RCTs, data was extracted from the included studies. We used a predefined Excel sheet for data extraction that incorporated the following variables: the trial registry, name of the primary author, year of publication, country of the study, design of the RCT, blinding status, total sample size, sample size of each arm, sex distribution, median age, dosage and duration of BDQ administration, other antituberculous medications used for each arm, and the timing of the culture follow-up. The distribution of resistance profiles for each study was also recorded: RR-TB (i.e., TB that is resistant to rifampin), MDR-TB (i.e., TB that is resistant to both isoniazid and rifampin), pre-XDR-TB (i.e., TB that is resistant to isoniazid, rifampicin, plus any fluoroquinolones), and XDR-TB (i.e., TB that is resistant to isoniazid, rifampin, any fluoroquinolone, plus at least one additional drug categorized as a group A drug [levofloxacin/moxifloxacin, BDQ, or linezolid] OR a second-line injectable agent [amikacin, kanamycin, and capreomycin]) [14]. Furthermore, data on safety and efficacy were extracted.

### 2.4. Quality Assessment

Two reviewers (IS and YS) independently assessed the quality of all included RCTs using the Jadad scale, which includes the following three domains: randomization (scored 0, 1, or 2 points), double-blinding (scored 0, 1, or 2 points), and adequate reporting of withdrawals and dropouts (scored 0 or 1 point) [15]. The total possible score ranges from 0 to 5 points. High-quality RCTs were those with a total score of ≥ 3, whereas those with a score of < 3 were considered low-quality studies.

### 2.5. Outcome Measures

Safety outcomes incorporated all-cause mortality, nonserious adverse effects (AEs), and serious AEs (SAEs). Efficacy outcome measures included culture conversion rate at 8–12 and 24–26 weeks, treatment success (defined as the sum of both cured and treatment completed), and time to culture conversion (in days). The definition of each treatment outcome was in accordance with the 2013 WHO outcome definition for DR-TB, which was also consistent with the definitions in other published studies [10, 12, 13, 16].

### 2.6. Statistical Analysis

Safety outcome measures (i.e., all-cause mortality, AEs, and SAEs) were regarded as dichotomous variables and were reported as relative risk (RR) with a 95% confidence interval (CI). Similarly, both early culture conversion rates and treatment success were described as dichotomous variables and reported as RR with a 95% CI. On the other hand, time to sputum culture conversion was a continuous variable (in days) and was reported as a standardized mean difference (SMD). A random-effect model was used owing to a variety of population origins. Heterogeneity between studies was evaluated using *I*^2^ statistics, with the *I*^2^ values of < 25%, 25%–75%, and > 75% considered as low, moderate, and high degrees of heterogeneity, respectively.

Publication bias was evaluated through a funnel plot and Egger's regression test. In addition, standard leave-one-out sensitivity analyses were conducted for both safety and efficacy outcomes to identify the studies that exerted the most significant influence on the pooled estimates. A *p* value of less than 0.05 was considered statistically significant. All statistical analyses were performed using both the IBM SPSS Statistics for Mac (Version 28.0, IBM Corp., Armonk, NY) and Stata (Stata Statistical Software: Version 17, College Station, TX: StataCorp LP).

## 3. Results

### 3.1. Literature Search Results

**Figure 1:**
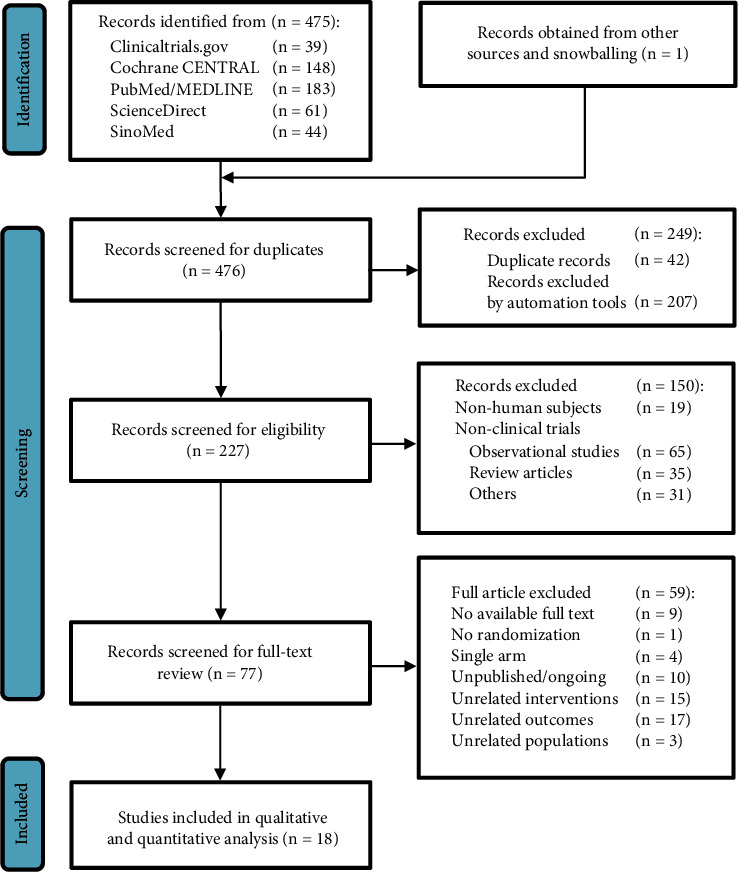
PRISMA flow chart.

Figure 1 summarizes the selection of studies. A total of 476 studies were identified from five databases, i.e., ClinicalTrials.gov (*n* = 39), Cochrane CENTRAL (*n* = 148), PubMed/MEDLINE (*n* = 183), ScienceDirect (*n* = 61), and SinoMed (*n* = 44). One study was retrieved through snowballing. After the initial screening for duplicate records (*n* = 42) and using automated screening tools for each database (*n* = 207), a title and abstract screening further excluded 150 studies (observational studies [*n* = 65], review articles [*n* = 35], irrelevant studies [*n* = 31], and animal studies [*n* = 19]). The final screening of full texts excluded studies with unrelated outcomes (*n* = 17) or unrelated intervention (*n* = 15), unpublished or ongoing trials (*n* = 10), studies whose full text was unavailable (*n* = 9), and studies with single-arm intervention (*n* = 4), unrelated populations (*n* = 3), or an absence of randomization (*n* = 1). A total of 18 studies were included in both the qualitative and quantitative analyses.

### 3.2. Characteristics of Included Studies

The included RCTs were published between 2012 and 2023. The total pooled sample size was 2520 participants, 55.8% of whom received BDQ-containing regimens (*n* = 1408). The ratio of men to women was approximately 1.3–1.0. The majority of RCTs were open-label trials (*n* = 9), and only two studies reported blinding [8, 17]. The dose of oral BDQ was generally consistent among the majority of trials, with an initial dose of 400 mg per day for the first 2 weeks followed by 200 mg for up to 22–26 weeks. Three RCTs extended the BDQ-containing regimen beyond 30 weeks [18–20]. Furthermore, sputum culture follow-up was performed at different times, ranging from 2 to 120 weeks after the initiation of treatment, but the majority were performed between Weeks 24 and 26. The characteristics of the included RCTs are summarized in Table 1.

Among the participants, patients with RR-TB constituted the largest cohort (*n* = 1303), followed by MDR-TB (*n* = 1142). Pre-XDR-TB was reported in a smaller population from a single study (*n* = 27), and there were no documented cases of XDR-TB in the included RCTs (Supporting Information: Appendix 1).

### 3.3. Quality of Studies

The quality of each included RCTs was assessed using the Jadad scoring system. The summary of the quality assessment is in Supporting Information: Appendix 2. The studies' Jadad scores ranged from 1 to 3 points. Thirteen studies were of high quality (all scored 3 points) [8, 17, 18, 20, 22, 23, 25, 27–30, 32], whereas five studies were considered low quality (four studies scored 2 points [19, 24, 26, 31]); one study scored 1 point [21]).

### 3.4. Outcome Analysis

#### 3.4.1. All-Cause Mortality, SAEs, and AEs

**Figure 2:**
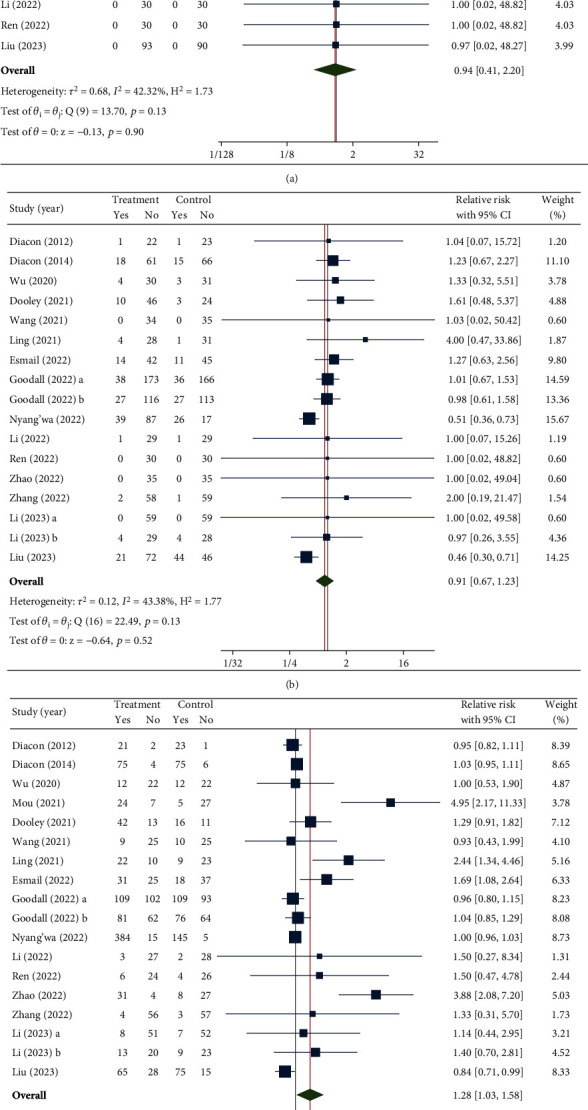
Forest plot of safety outcomes, including (a) all-cause mortality, (b) serious adverse events, and (c) adverse events.

Ten studies reported data on all-cause mortality. As illustrated in Figure 2(a), there was an insignificant pooled mortality benefit in the BDQ arm (RR [95%CI] = 0.94 [0.41–2.20], *p* value = 0.90). Similarly, the rate of SAE reduction, as analyzed by 17 RCTs, was insignificant (RR [95%CI] = 0.91 [0.67–1.23], *p* value = 0.52) (Figure 2(b)). There was a significant rise in AEs in the BDQ arm compared to the controls (RR = 1.28 [95% CI: 1.03–1.58], *p* value = 0.02) (Figure 2(c)). The leave-one-out sensitivity analysis revealed a nonsignificant rise in the rate of AEs when omitting an RCT by either Houling [21] (RR [95%CI] = 1.18 [0.99–1.40]), Huiqi, Weijie, and Xianke [24] (RR [95%CI] = 1.22 [1.00–1.49]), or Shan and Jian [28] (RR [95%CI] = 1.14 [0.98–1.32]) (Supporting Information: Appendix 3). In addition, moderate between-study heterogeneity was observed in both the all-cause mortality (*I*^2^ = 42.32%) and SAE endpoints (*I*^2^ = 43.38%), whereas the AE endpoint demonstrated high heterogeneity (*I*^2^ = 94.10%).

#### 3.4.2. Culture Conversion Rates, Treatment Success, and Time to Culture Conversion

**Figure 3:**
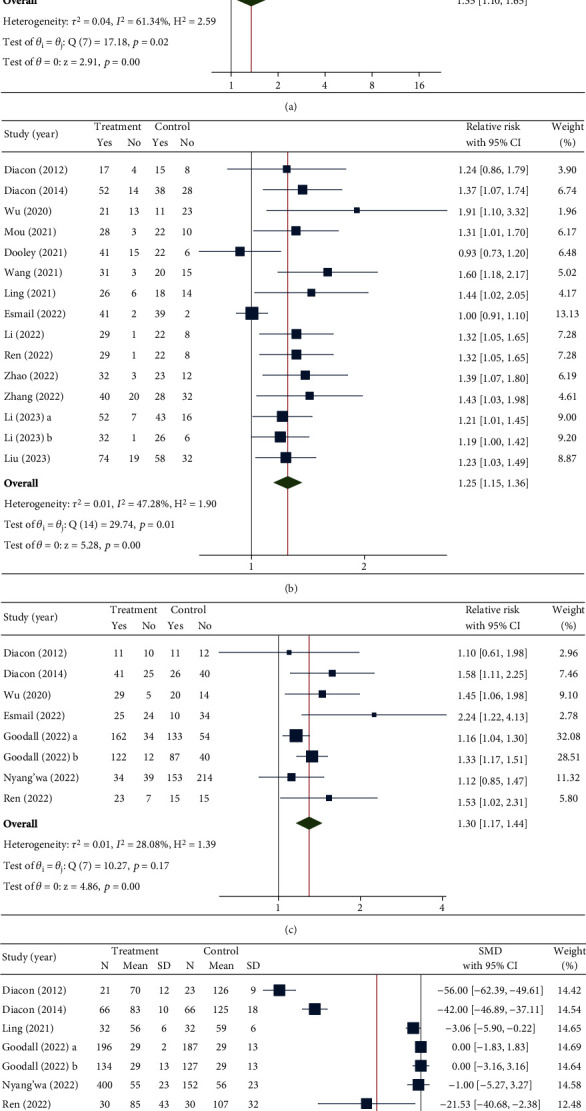
Forest plot of efficacy outcomes, including sputum culture conversion rate at (a) 8–12 weeks and at (b) 24–26 weeks, (c) treatment success, and (d) time to culture conversion (days). SMD, standardized mean difference.

The rate of sputum culture conversion at 8–12 and 24–26 weeks was analyzed in eight and 15 RCTs, respectively. At Weeks 8–12, there was a significant difference in the culture conversion rate between the two arms (RR [95%CI] = 1.35 [1.10–1.65], *p* value < 0.001), favoring the BDQ arm (Figure 3(a)). Furthermore, as shown in Figure 3(b), the analysis at 24–26 weeks revealed a significant 25% increase in the probability of culture conversion in the BDQ arm compared to the controls (RR [95%CI] = 1.25 [1.15–1.36], *p* value < 0.001). Both study endpoints demonstrated a moderate degree of heterogeneity across RCTs, with *I*^2^ = 61.34% for the culture conversion rate at Weeks 8–12 and *I*^2^ = 47.28% at Weeks 24–26.

Treatment success was evaluated in eight RCTs (Figure 3(c)) and showed a significant 30% increase in the rate of favorable outcomes compared to the control group (RR [95%CI] = 1.30 [1.17–1.44], *p* value < 0.001). In addition, for time to culture, a pooled SMD from seven RCTs showed approximately 17 fewer days to culture conversion compared to the controls (SMD [95%CI] = −17.46 [−34.82 to −0.11], *p* value = 0.05) (Figure 3(d)). The heterogeneity of the treatment success outcome across studies was low (*I*^2^ = 28.08%), whereas between-study heterogeneity for time to culture conversion was high (*I*^2^ = 99.37%).

### 3.5. Publication Bias Assessment

The funnel plots of all study endpoints are shown in Supporting Information: Appendix 4. For the safety outcomes, a regression-based Egger's test for small-study effects demonstrated a low risk of publication bias for all-cause mortality (*p* value = 0.98) and SAEs (*p* value = 0.16), whereas AEs demonstrated a high risk of publication bias (*p* value < 0.001). On the other hand, efficacy endpoints invariably demonstrated a high-risk publication bias in culture conversion at 8–12 weeks (*p* value < 0.001) and 24–26 weeks (*p* value < 0.001) and in time to culture conversion (*p* value < 0.001). The treatment success endpoints demonstrated a low-risk publication bias (*p* value = 0.07).

## 4. Discussion

The treatment strategy for DR-TB has evolved throughout the decades [33]. Most recently, the 2019 WHO Consolidated Guidelines on Drug-Resistant TB reclassified antituberculosis agents into groups A, B, and C; BDQ was incorporated into group A along with linezolid and moxifloxacin/levofloxacin [34]. As treatment with group A drugs should be prioritized whenever possible, BDQ has become one of the most important regimens to combat DR-TB infections. This systematic review and meta-analysis assessed 18 RCTs to evaluate the safety and efficacy of BDQ-containing regimens in the treatment of DR-TB. Overall, the findings suggest no difference in all-cause mortality or SAEs; AEs were significantly higher in the BDQ-receiving arm. Furthermore, BDQ-containing regimens significantly improved both early (8–12 weeks) and late (24–26 weeks) culture conversion rates and treatment success, and the time to culture conversion was accelerated by 17 days.

This is the first study that has attempted to evaluate pooled evidence of the safety and efficacy of BDQ in the treatment of DR-TB based exclusively on available data from RCTs. Our findings suggest an insignificant mortality benefit of BDQ-containing regimens. At the time of writing this meta-analysis, two published meta-analyses have examined pooled evidence on mortality based on mostly observational studies, both of which contradicted our finding [10, 11]. A review by Wang, Wu, and He [10] involving eight studies, two of which were RCTs, demonstrated a significant 47% reduction in mortality risk in the BDQ-treatment arm. Their finding was consistent with a review by Rehman et al. [13] and a subgroup analysis of pooled observational studies by Tong et al. [11], which demonstrated a significant mortality risk reduction of 27% and 32%, respectively. In contrast, the pooled analysis of five RCTs by Tong et al. [11] demonstrated a nonsignificant increase in mortality. This discrepancy and inconsistency in mortality benefit findings may be attributed to the inclusion of discrete categories of studies in each review. However, it is evident that the inclusion of more RCTs in our study has resulted in an insignificant reduction in mortality.

All 18 included RCTs reported data on AEs, whereas only 17 trials reported on SAEs. Importantly, patients treated with BDQ exhibited a significant 28% higher risk of nonserious AEs compared to the control group. However, most AEs are considered mild to moderate and do not necessitate the discontinuation of BDQ-containing regimens [35]. Other drugs in DR-TB regimens, however, may contribute to various accompanying AEs [17, 19]. Unlike the finding of a significantly higher risk of AEs in the BDQ arm, our study found a nonsignificant difference in SAEs between the two arms. This is in contrast to the review by Tong et al. that reported a significant 42% higher probability of Grade 3–5 AEs in the BDQ group compared to the control group [11]. This discrepancy might be due to variations in sample characteristics; Tong et al. [11] included mostly observational studies. In addition, there may have been differences in how AEs were classified and defined by the individual studies.

Our analysis revealed significantly higher culture conversion rates with BDQ-containing regimens at both 8–12 weeks and 24–26 weeks by 35% and 25%, respectively. This is consistent with other studies by Wang, Wu, and He [10] and Tong et al. [11], who also noted improvement in the overall culture conversion rate. Moreover, the RCT subanalysis by Tong et al. [11] of the culture conversion rate at 24 weeks was 27% higher among the BDQ-regimen group. We also found that the treatment success rate was significantly higher by 30% among the BDQ-containing regimen arm. Apart from Wang, Wu, and He [10], all previous systematic reviews [11–13] reported significant improvement in the treatment success rate.

The current conditional recommendation in the updated 2022 WHO Consolidated Guidelines on Tuberculosis suggests a 6-month treatment with a combination of BDQ, pretomanid, linezolid (600 mg), and moxifloxacin, known as the BpaLM regimen, for RR-TB, MDR-TB, and pre-XDR-TB (quality of evidence: very low) [36]. This is shorter than the previously recommended 9 months or longer duration [34, 36]. The guidelines suggest that the initiation should not be delayed by the drug susceptibility testing (DST) for fluoroquinolones. The moxifloxacin, however, should be stopped if fluoroquinolone resistance is proven by a DST (i.e., use a BPaL regimen instead of a BpaLM regimen). Available data from the 2-stage phase 2/3 TB-PRACTECAL trial showed higher treatment success rates with BPaLM compared to standard of care regimens (89% vs. 52% among patients with RR-TB) [25]. The BPaLM regimen also had superior treatment success compared to BPaL and BPaLC (i.e., BPaL plus clofazimine) regimens (89% vs. 81% and 77%, respectively) [25].

Apart from the published TB-PRACTECAL, ZeNIX, and NIX-TB trials that incorporated BDQ (especially in the BPaL or BpaLM formulation regimens) [25, 37, 38], several other clinical trials are also ongoing. These include the following from clinicaltrials.gov registries:

• NCT05381194: BPaL[M] regimen for the treatment of MDR/RR-TB
• NCT05040126: modified BPaL regimen for managing pre-XDR TB and MDR (TI/NR) TB in India (mBPaL)
• NCT06058299: Phase 2 trial assessing TBAJ876 or BDQ, with pretomanid and linezolid in adults with drug-sensitive pulmonary tuberculosis

These pursuits toward establishing the risks and benefits of these combination drugs are a positive trend that will advance the use of BDQ and help fight against DR-TB for decades to come.

### 4.1. Strengths and Limitations

Our meta-analysis consisted exclusively of prospective clinical trials with the appropriate controlled arms, and during the Jadad score assessment, we eliminated any study that did not apply a randomization strategy. We also incorporated an additional Chinese database, that is, SinoMed, into our search strategy, which resulted in the inclusion of 11 more RCTs (61% of total included RCTs). This eliminated the language barrier limitation and considerably improved the pooled analysis in our study. We performed analyses on early culture conversion (Weeks 8–12) and culture conversion after the BDQ intensive phase ended (Weeks 24–26). The findings in our study clearly point toward the superiority of combining BDQ with other second-line agents.

However, this study had several limitations. The limited data on each resistance profile in the individual RCTs, that is, RR-TB, MDR-TB, pre-XDR-TB, and XDR-TB, limited the subgroup analyses for both the safety and efficacy outcomes. There was also insufficient data on pregnant women, children, and individuals with human immunodeficiency virus (HIV) infection and with chronic liver, cardiac, and renal comorbidities. Furthermore, the lack of blinding in the majority of the BDQ RCTs and its effect on the studies' quality may also have affected the reliability of the pooled estimates in this review. Finally, the analyses on AEs and SAEs did not specify which organ systems were affected. Not enough is known about BDQ's cardiotoxic side effects, particularly its proarrhythmogenic potential to widen the QT interval. Future studies should explore pooled data from clinical trials on arrhythmogenic side effects of BDQ.

## 5. Conclusion

This review provides strong evidence for the efficacy and safety of BDQ-containing regimens in the treatment of DR-TB. Patients receiving BDQ had significantly higher rates of treatment success and culture conversion both at Weeks 8–12 and Weeks 24–26 compared to BDQ-free patients. This suggests better success in the eradication of TB infections. Although some AEs were more common with BDQ, they were not significantly more severe. However, limitations like limited data on specific resistance profiles and the potential for proarrhythmogenic effects highlight the need for further studies. Overall, this study strengthens the evidence for administering BDQ in the fight against DR-TB.

## Figures and Tables

**Table 1:** Baseline characteristics of the included RCTs.

**Author (year)**	**Country**	**Study design, phase, blinding**	**Σ** **(** **n** **)**	**Arm**	**Each arm (** **n** **)**	**Sex (** **n** **)**		**Median (SD) age**	**Intervention**	**BDQ dose (mg)**	**Other anti-tuberculous drug regimens**	**Duration of BDQ Use**	**Sputum culture follow-up (week)**
						**M**	**F**						
Diacon (2012) [8]	Multiple countries^a^	RCT, II, triple-blind	47	Tx	23	18	5	35.6 (11.66)	BDQ	400 qd (2 w), then 200 tiw (6 w)	Eto, Kan, Ofl, E/Trd-Cyc/Dap	8	8, 24, 104
				Con	24	17	7	33.6 (11.04)	Placebo		Eto, Kan, Ofl, E/Trd-Cyc/Dap	—	

Diacon (2014) [17]	Multiple countries^a^	RCT, II, triple-blind	160	Tx	79	52	27	36.2 (13.13)	BDQ	400 qd (2 w), then 200 tiw (22 w)	Eto, Kan, Ofl, Z, Cyc	24	24, 72, 120
				Con	81	49	32	35.8 (11.01)	Placebo		Eto, Kan, Ofl, Z, Cyc	—	

Haoyu (2020) [18]	China	RCT, II, NA	68	Tx	34	19	15	41.02 (3.55)	BDQ	400 qd (2 w), then 200 qd (22–46 w)	H, E, Z	24-46	12, 24, 48
				Con	34	20	14	40.11 (3.82)	No BDQ		H, E, Z	—	

Houling (2021) [21]	China	RCT, II, open-label	63	Tx	31	24	7	34.25 (2.01)	BDQ	400 qd (2 w), then 200 tiw (22 w)	Mfx, Pto, Z, Cs, Lzd	24	24
				Con	32	21	11	34.35 (1.95)	No BDQ		Mfx, Pto, Z, Cs, Lzd	—	

Dooley (2021) [22]	South Africa, Peru	RCT, II, open-label	84	Tx	56	42	14	34.25 (16.6)	BDQ	400 qd (2 w), then 200 tiw (22 w)	Std ± Dlm	24	2-weekly
				Con	28	21	7	32 (25)	Dlm		Std (no Cfz)	—	

Fei (2021) [23]	China	RCT, II, open-label	69	Tx	34	18	16	54.1 (7.3)	BDQ	400 qd (2 w), then 200 tiw (24 w)	H, E, Z	26	26
				Con	35	19	16	52.7 (6.8)	No BDQ		H, E, Z, Mox	—	
						**M**	**F**						

Huiqi, Weijie, and Xianke (2021) [24]	China	RCT, II, NA	64	Tx	32	18	14	45.35 (4.61)	BDQ	400 qd (2 w), then 200 tiw (22 w)	Z, Pth, E, Mox, Cyc	24	6, 12, 24
				Con	32	19	13	45.47 (4.59)	No BDQ		Z, Pth, E, Mox, Cyc	—	

Esmail (2022) [19]	South Africa	RCT, II/III, open-label	93	Tx	49	34	15	37 (4.9)	BDQ	400 qd (2 w), then 200 tiw (24–36 w)	Lzd, Lev, Z, Trd/Eto/H	24-36	60–72
				Con	44	28	16	36 (7.2)	No BDQ		Kan, Mox, Cfz, Z, E, Trd/Eto/H	—	

Goodall et al. (2022) a [20]	Multiple countries^b^	RCT, III, NA	383	Tx	196	124	72	32.8 (6.57)	BDQ	400 qd (2 w), then 200 tiw (40 w)	Cfz, E, Z, H, Pth, Lev	40	4-weekly (w52), then 8-weekly (w76
				Con	187	115	72	32.8 (6.14)	No BDQ		Cfz, E, Z, H, Pth, Mox/Lev, Kan	—	

Goodall et al. (2022) b [20]	Multiple countries^b^	RCT, III, NA	261	Tx	134	81	53	30.8 (6.73)	BDQ	400 qd (2 w), then 200 tiw (28 w)	Lev, Cfz, Z, H, Kan	28	4-weekly (w52), then 8-weekly (w76
				Con	127	77	50	37.0 (6.76)	No BDQ		Std	—	

Nyang'wa et al. (2022) [25]	Belarus, South Africa, Uzbekistan	RCT, II–III, open-label	552	Tx	400	166	234	34 (24.5)	BDQ	400 qd (2 w), then 200 tiw (22 w)	Pmd, Lzd, Mox, Cfz	24	8
				Con	152	96	56	37 (22.9)	No BDQ		Std	—	

Yanli and Weijuan (2022) [26]	China	RCT, II, open-label	60	Tx	30	17	13	57.82 (11.13)	BDQ	400 qd (2 w), then 200 tiw (22 w)	Mfx, Am, Pto, PAS	24	12, 24
				Con	30	16	14	58.21 (11.22)	No BDQ		Mfx, Am, Pto, PAS	—	
						M	F						

Na et al. (2022) [27]	China	RCT, II, open-label	60	Tx	30	19	11	47.12 (12.33)	BDQ	400 qd (2 w), then 200 tiw (22 w)	Cm/Am, Lev/Mox, Pto/PAS/E, Cs/PAS/E	24	4, 8, 12, 24
				Con	30	17	13	46.85 (13.01)	No BDQ		Cm/Am, Lev/Mox, Pto/PAS/E, Cs/PAS/E	—	

Shan, Jian, and Chunjing (2022) [28]	China	RCT, II, open-label	70	Tx	35	23	12	34.33 (1.92)	BDQ	400 qd (2 w), then 200 tiw (22 w)	Mox, Pto, Z, Lzd, Cs	24	24
				Con	35	25	10	34.23 (1.97)	No BDQ		Mox, Pto, Z, Lzd, Cs	—	

Guofei (2022) [29]	China	RCT, II, open-label	120	Tx	60	37	23	48.73 (8.2)	BDQ	400 qd (2 w), then 200 tiw (22 w)	Lzd, Z, Pto, Mox	24	12, 24
				Con	60	39	21	48.12 (7.1)	No BDQ		Lzd, Z, Pto, Mox	—	

Jiangkang et al. (2023) a [30]	China	RCT, II, NA	118	Tx	59	35	24	43.83 (6.9)	BDQ	400 qd (2 w), then 200 tiw (24 w)	Z, Pth, Lev, Cyc-Lzd	26	26
				Con	59	33	26	43.25 (7.18)	No BDQ		Z, Pth, Lev, Cyc-Lzd	—	

Jinghong and Yuqing (2023) b [31]	China	RCT, II, open-label	65	Tx	33	21	12	35.44 (6.08)	BDQ	400 qd (2 w), then 200 tiw (22 w)	Lfx, Pto, Cs, Lzd	24	24
				Con	32	19	13	36.73 (5.62)	No BDQ		Lfx, Pto, Cs, Lzd	—	

Liu et al. (2023) [32]	China	RCT, II, open-label	183	Tx	93	58	35	42 (10.46)	BDQ	400 qd (2 w), then 200 tiw (22 w)	Lzd/Cyc/Mox/Ami/E/Lev/Pth/Str/Cfz	24	8, 24
				Con	90	63	27	37 (8.39)	No BDQ		Lzd/Cyc/Mox/Ami/E/Lev/Pth/Str/Cfz	—	

Abbreviations: Ami: amikacin; BDQ: bedaquiline; Cfz: clofazimine; Con: control arm; Cyc: cycloserine; Dap: dapsone; Dlm: delamanid; E: ethambutol; Eto: ethionamide; Flq: fluoroquinolone; H: isoniazid; Kan: kanamycin; Lev: levofloxacin; Lzd: linezolid; Mox: moxifloxacin; NA: not applicable; nRCT: nonrandomized controlled trial; Ofl: ofloxacin; PAS: para-aminosalicylic acid; Pmd: pretomanid; Pth: prothionamide; qd: quaque die (once a day); R: rifampin; RCT: randomized controlled trial; Std: standard regimen; Str: streptomycin; tiw: three times a week; Trd: terizidone; Tx: treatment arm; w: week; Z: pyrazinamide.

^a^Brazil, India, Latvia, Peru, Philippines, Russia, South Africa, and Thailand.

^b^Ethiopia, Georgia, India, Moldova, Mongolia, South Africa, and Uganda.

## Data Availability

Data from this study is available from the corresponding author upon reasonable request.

## Supporting Information

Supporting Information Additional supporting information can be found online in the Supporting Information section. Appendix 1 Table S1: tuberculosis resistance profiles in the included RCTs. Appendix 2: Table S2: summary of the Jadad score for the included studies. Appendix 3: Figure S1: leave-one-out sensitivity analysis of all safety and efficacy outcomes, including (A) all-cause mortality, (B) serious adverse events, (C) adverse events, (D) sputum culture conversion rate at 8–12 weeks and (E) at 24–26 weeks, (F) treatment success, and (G) time to culture conversion in days. Appendix 4: Figure S2: funnel plots of safety and efficacy outcomes. Regression-based Egger test for small-study effects using an inverse-variance method for (A) all-cause mortality (*p* value = 0.9830), (B) SAEs (*p* value = 0.1562), (C) AEs (*p* value ≤ 0.001), (D) sputum culture conversion at 8–12 weeks (*p* value ≤ 0.001), (E) sputum culture conversion at 24–26 weeks (*p* value < 0.001), (F) treatment success (*p* value = 0.0723), and (G) time to culture conversion (*p* value < 0.001).
