# Supplementary material for: Establishing the Safety and Efficacy of Bedaquiline-Containing Regimen for the Treatment of Drug-Resistant Tuberculosis: A Systematic Review and Meta-Analysis of Randomized Clinical Trials
Source: Pulm Med. 2024 Aug 9;2024:5542658. doi: 10.1155/2024/5542658 (PMC11329311; doi:10.1155/2024/5542658)
Supplement: Supporting Information — Additional supporting information can be found online in the Supporting Information section. Appendix 1 Table S1: tuberculosis resistance profiles in the included RCTs. Appendix 2: Table S2: summary of the Jadad score for the included studies. Appendix 3: Figure S1: leave-one-out sensitivity analysis of all safety and efficacy outcomes, including (A) all-cause mortality, (B) serious adverse events, (C) adverse events, (D) sputum culture conversion rate at 8–12 weeks and (E) at 24–26 weeks, (F) treatment success, and (G) time to culture conversion in days. Appendix 4: Figure S2: funnel plots of safety and efficacy outcomes. Regression-based Egger test for small-study effects using an inverse-variance method for (A) all-cause mortality (p value = 0.9830), (B) SAEs (p value = 0.1562), (C) AEs (p value ≤ 0.001), (D) sputum culture conversion at 8–12 weeks (p value ≤ 0.001), (E) sputum culture conversion at 24–26 weeks (p value < 0.001), (F) treatment success (p value = 0.0723), and (G) time to culture conversion (p value < 0.001). [file 5542658.f1.docx]

**SUPPLEMENTARY MATERIAL**

## **Appendix 1**

## **Table S1** Tuberculosis resistance profiles in the included RCTs.

| **Author (year)** | **Arm** | **TB Resistance Profile (n)** | | | | **Total (n)** |
| --- | --- | --- | --- | --- | --- | --- |
|  |  | **RR-TB** | **MDR-TB** | **Pre-XDR-TB** | **XDR-TB** |  |
| Diacon (2012) [8] | **Tx** |  | 23 |  |  | 23 |
|  | **Con** |  | 24 |  |  | 24 |
| Diacon (2014) [17] | **Tx** |  | 39 | 15 |  | 54 |
|  | **Con** |  | 46 | 12 |  | 58 |
| Wu (2020) [18] | **Tx** |  | 34 |  |  | 34 |
|  | **Con** |  | 34 |  |  | 34 |
| Mou (2021) [21] | **Tx** |  | 31 |  |  | 31 |
|  | **Con** |  | 32 |  |  | 32 |
| Dooley (2021) [22] | **Tx** | 56 |  |  |  | 56 |
|  | **Con** | 28 |  |  |  | 28 |
| Wang (2021) [23] | **Tx** |  | 34 |  |  | 34 |
|  | **Con** |  | 35 |  |  | 35 |
| Ling (2021) [24] | **Tx** |  | 32 |  |  | 32 |
|  | **Con** |  | 32 |  |  | 32 |
| Esmail (2022) [19] | **Tx** | 10 | 39 |  |  | 49 |
|  | **Con** | 13 | 31 |  |  | 44 |
| Goodall *a* (2022) [20] | **Tx** | 196 |  |  |  | 196 |
|  | **Con** | 187 |  |  |  | 187 |
| Goodall *b* (2022) [20] | **Tx** | 134 |  |  |  | 134 |
|  | **Con** | 127 |  |  |  | 127 |
| Nyang’wa (2022) [25] | **Tx** | 400 |  |  |  | 400 |
|  | **Con** | 152 |  |  |  | 152 |
| Li (2022) [26] | **Tx** |  | 30 |  |  | 30 |
|  | **Con** |  | 30 |  |  | 30 |
| Ren (2022) [27] | **Tx** |  | 30 |  |  | 30 |
|  | **Con** |  | 30 |  |  | 30 |
| Zhao (2022) [28] | **Tx** |  | 35 |  |  | 35 |
|  | **Con** |  | 35 |  |  | 35 |
| Zhang (2022) [29] | **Tx** |  | 60 |  |  | 60 |
|  | **Con** |  | 60 |  |  | 60 |
| Li (2023) a [30] | **Tx** |  | 59 |  |  | 59 |
|  | **Con** |  | 59 |  |  | 59 |
| Li (2023) b [31] | **Tx** |  | 33 |  |  | 33 |
|  | **Con** |  | 32 |  |  | 32 |
| Liu (2023) [32] | **Tx** |  | 93 |  |  | 93 |
|  | **Con** |  | 90 |  |  | 90 |
| **Total (n)** | | **1,303** | **1,142** | **27** | **0** | **2,472** |

Con: control arm; MDR: multidrug resistant; MDR-TB: tuberculosis that is resistant to both isoniazid and rifampin; pre-XDR-TB: pre-extensively drug resistant tuberculosis that is resistant to isoniazid, rifampicin, plus any fluoroquinolones; RR-TB: tuberculosis that is resistant to rifampin; TB: tuberculosis; Tx: treatment arm; XDR: extensively drug resistant; XDR-TB: tuberculosis that is resistant to isoniazid, rifampin, any fluoroquinolone, plus at least one additional drug categorized as a Group A drug [levofloxacin/moxifloxacin, BDQ, or linezolid] OR a second-line injectable agent [amikacin, kanamycin, and capreomycin]).

## **Appendix 2**

## **Table S2.** Summary of the Jadad score for the included studies.

| **Studies** | **Jadad scale** | | | | **Quality** |
| --- | --- | --- | --- | --- | --- |
|  | **R**  **(0-2)** | **B**  **(0-2)** | **D**  **(0-1)** | **Total**  **(0-5)** |  |
| **Diacon (2012)** | 1 | 1 | 1 | 3 | High |
| **Diacon (2014)** | 1 | 1 | 1 | 3 | High |
| **Wu (2020)** | 2 | 0 | 1 | 3 | High |
| **Mou (2021)** | 1 | 0 | 0 | 1 | Low |
| **Dooley (2021)** | 2 | 0 | 1 | 3 | High |
| **Wang (2021)** | 2 | 0 | 1 | 3 | High |
| **Ling (2021)** | 1 | 0 | 1 | 2 | Low |
| **Esmail (2022)** | 1 | 0 | 1 | 2 | Low |
| **Goodall (2022) a** | 2 | 0 | 1 | 3 | High |
| **Goodall (2022) b** | 2 | 0 | 1 | 3 | High |
| **Nyang’wa (2022)** | 2 | 0 | 1 | 3 | High |
| **Li (2022)** | 1 | 0 | 1 | 2 | Low |
| **Ren (2022)** | 2 | 0 | 1 | 3 | High |
| **Zhao (2022)** | 2 | 0 | 1 | 3 | High |
| **Zhang (2022)** | 2 | 0 | 1 | 3 | High |
| **Li (2023) a** | 2 | 0 | 1 | 3 | High |
| **Li (2023) b** | 2 | 0 | 0 | 2 | Low |
| **Liu (2023)** | 2 | 0 | 1 | 3 | High |

B: blinding; D: drop-out; R: randomization

# **Appendix 3.**

# **All-cause mortality**


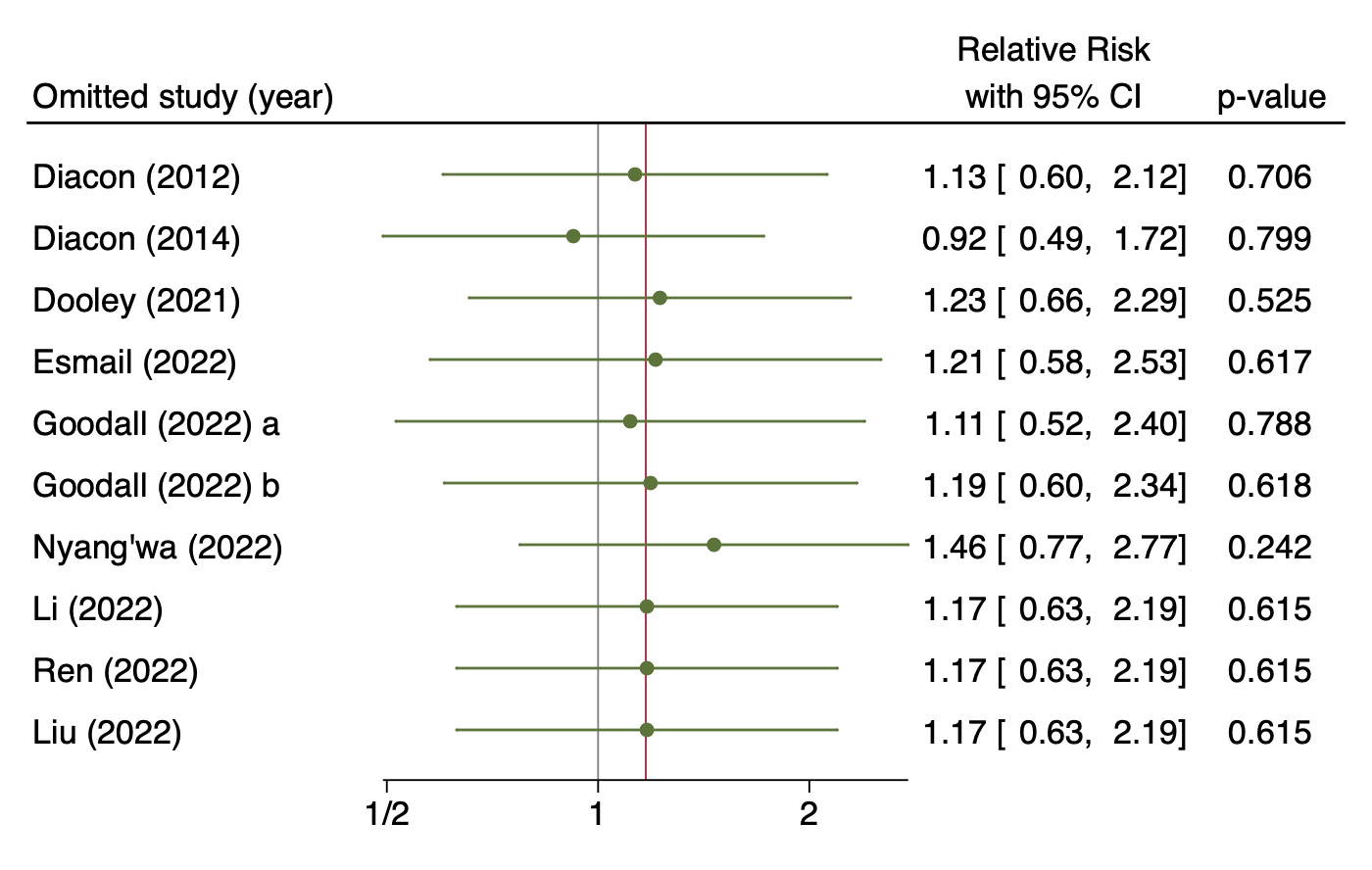


1. **Serious adverse events**


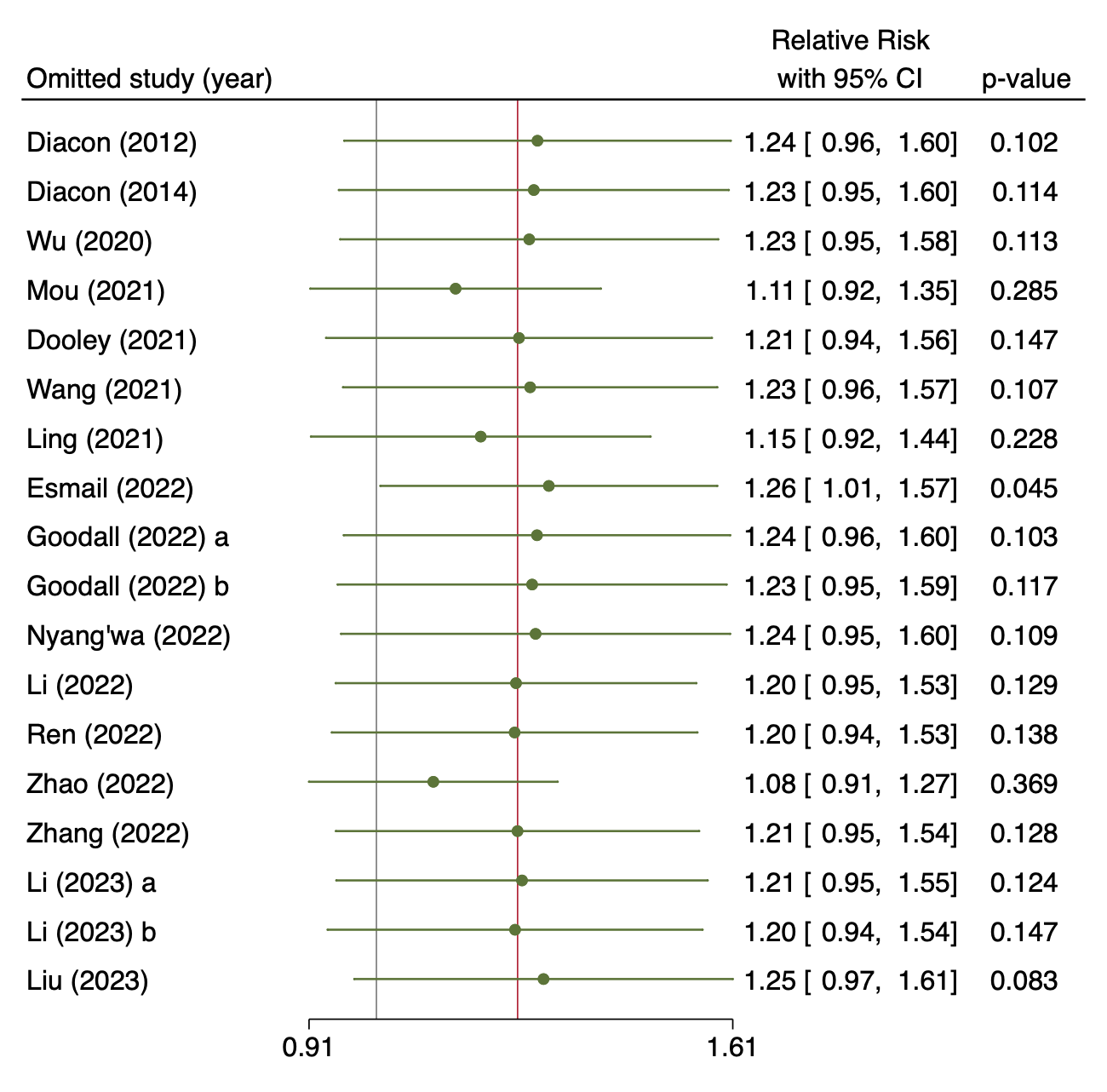


## **Non-serious adverse events**


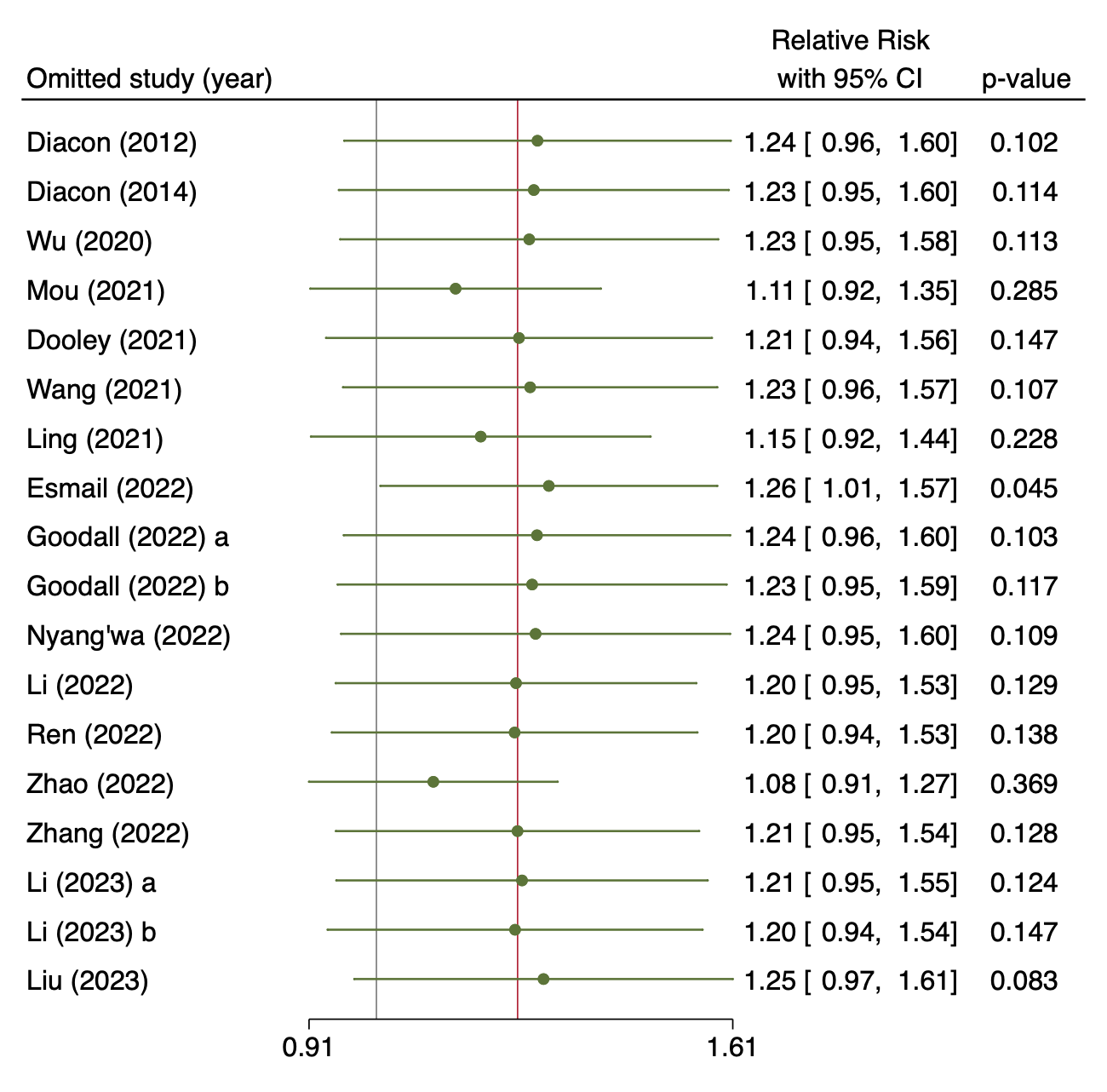


##

## **Culture Conversion at Week 8-12**


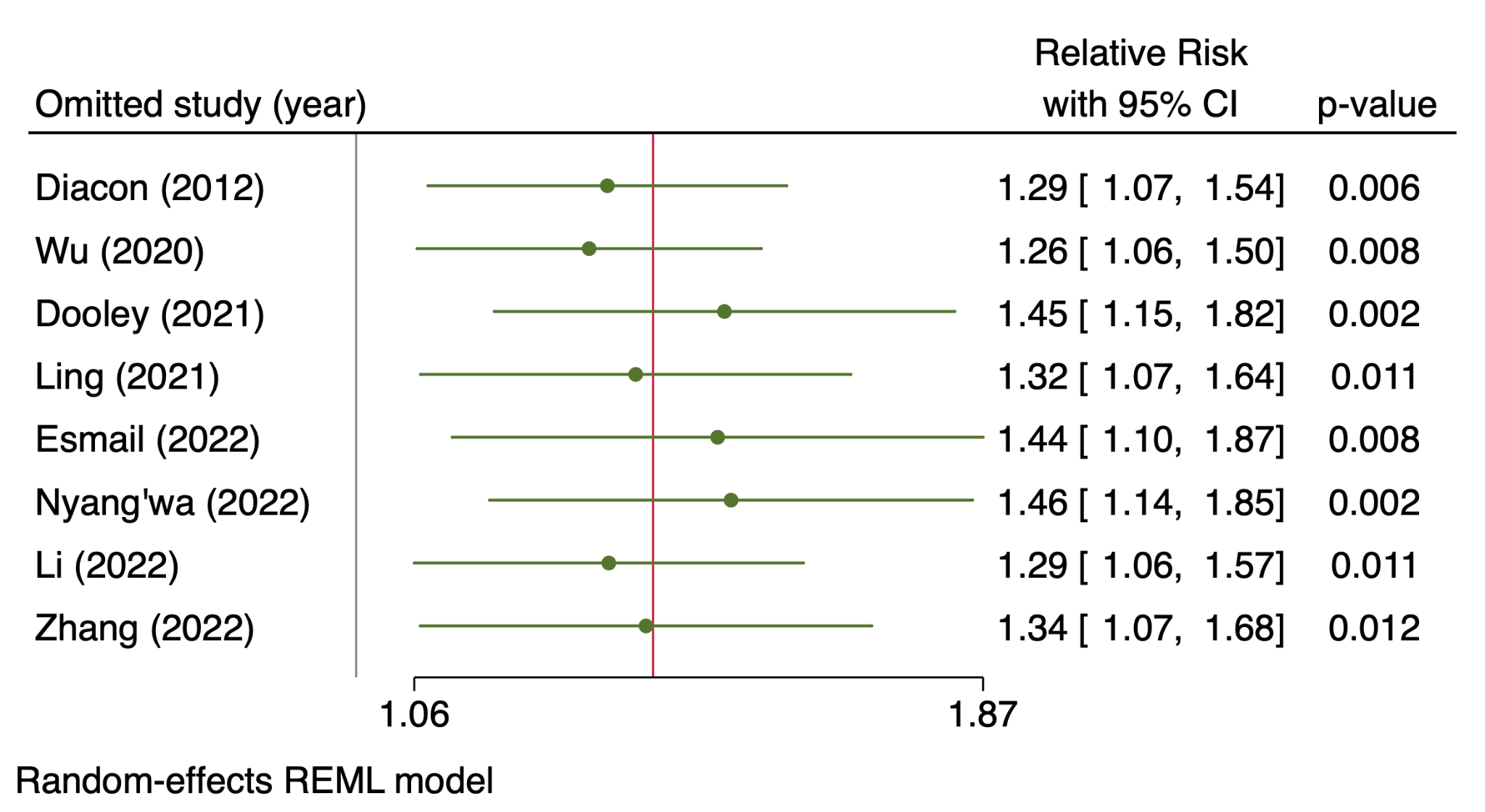


## **Culture Conversion at Week 24-26**


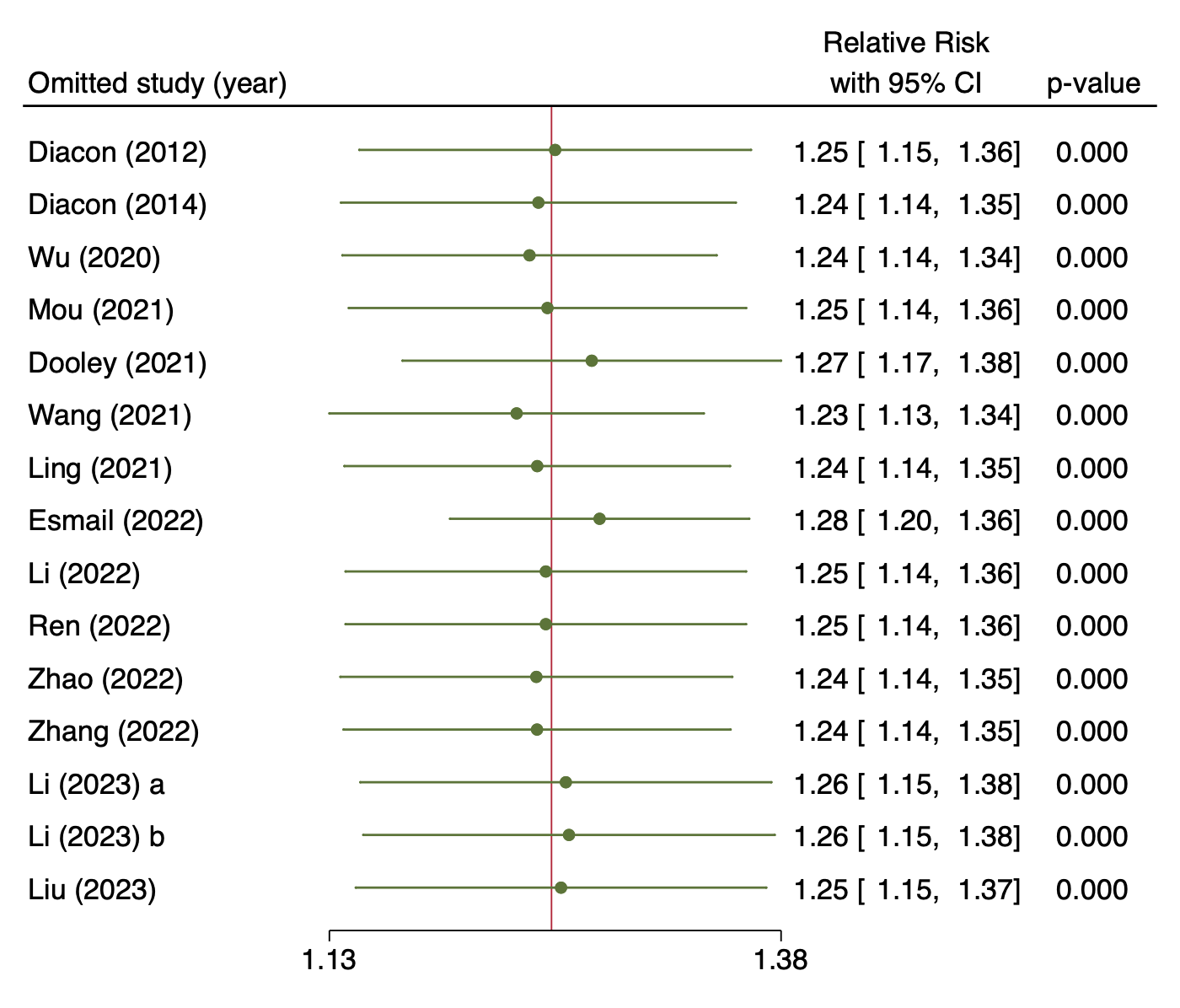


1. **Treatment success**


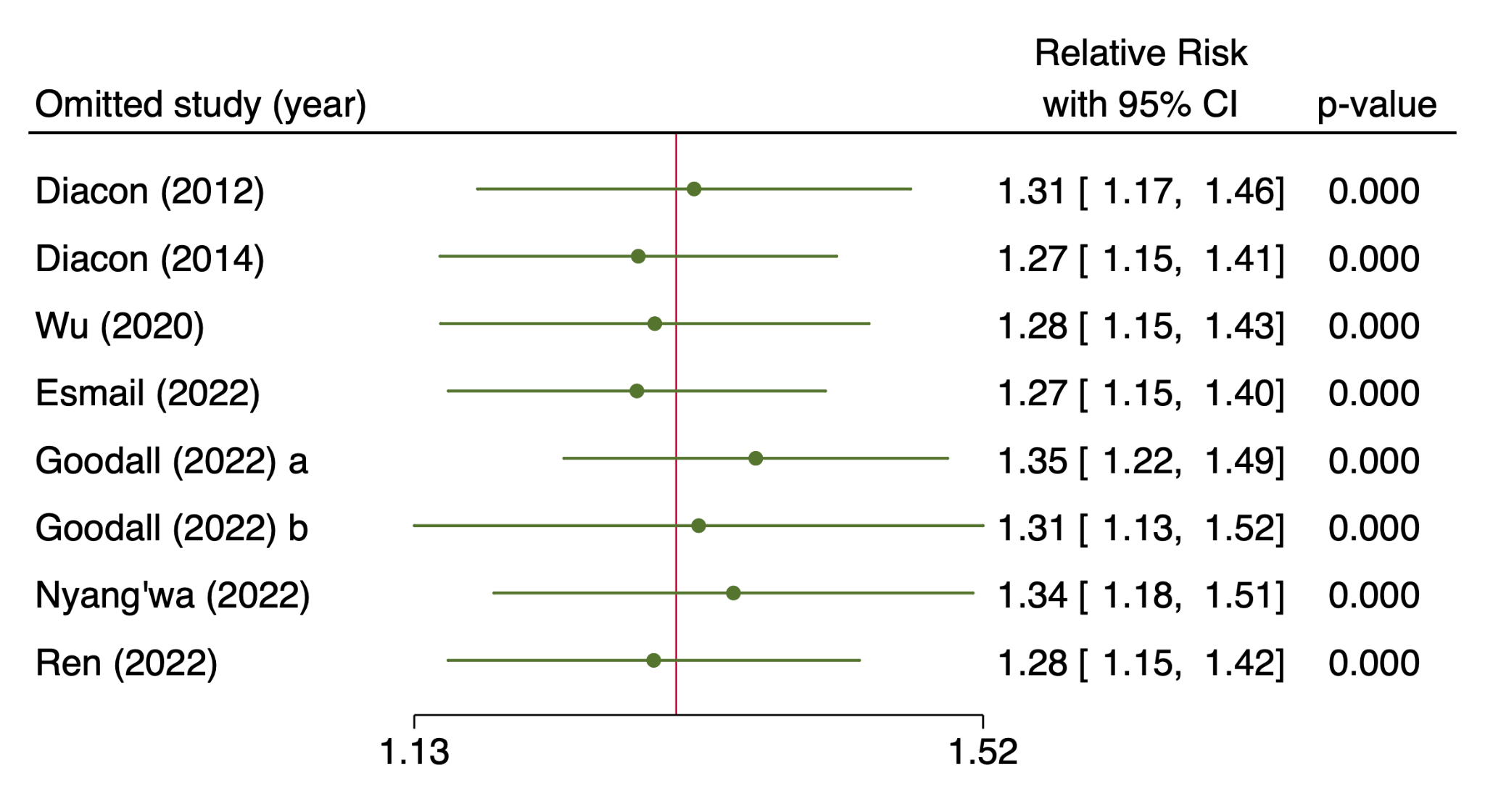


##

## **Time to Culture Conversion**


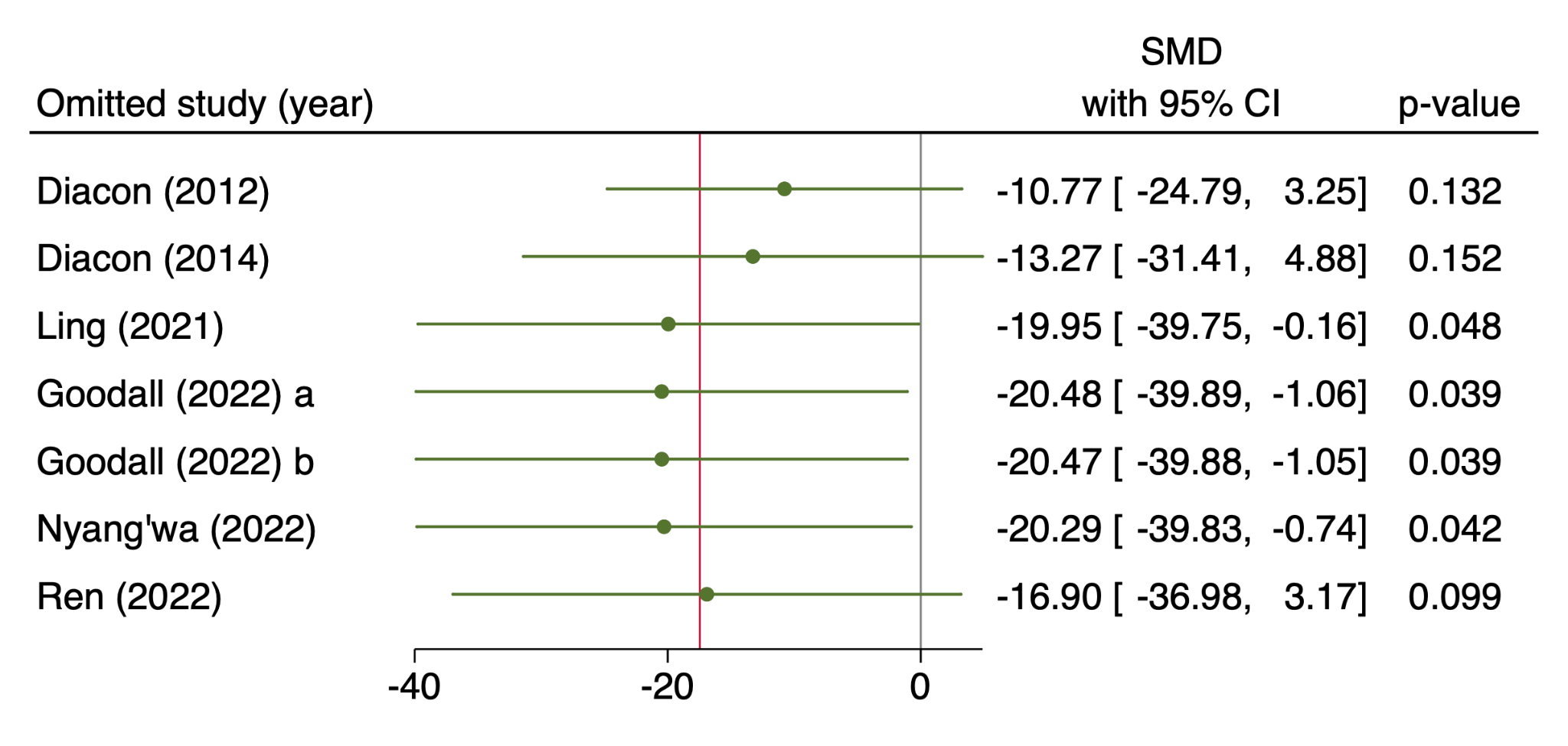


# **Figure S1.** Leave-one-out sensitivity analysis of all safety and efficacy outcomes, including (A) all-cause mortality, (B) serious adverse events, (C) adverse events, (D) sputum culture conversion rate at 8-12 weeks and (E) at 24-26 weeks, (F) treatment success, and (G) time to culture conversion in days.

**Appendix 4.**

| **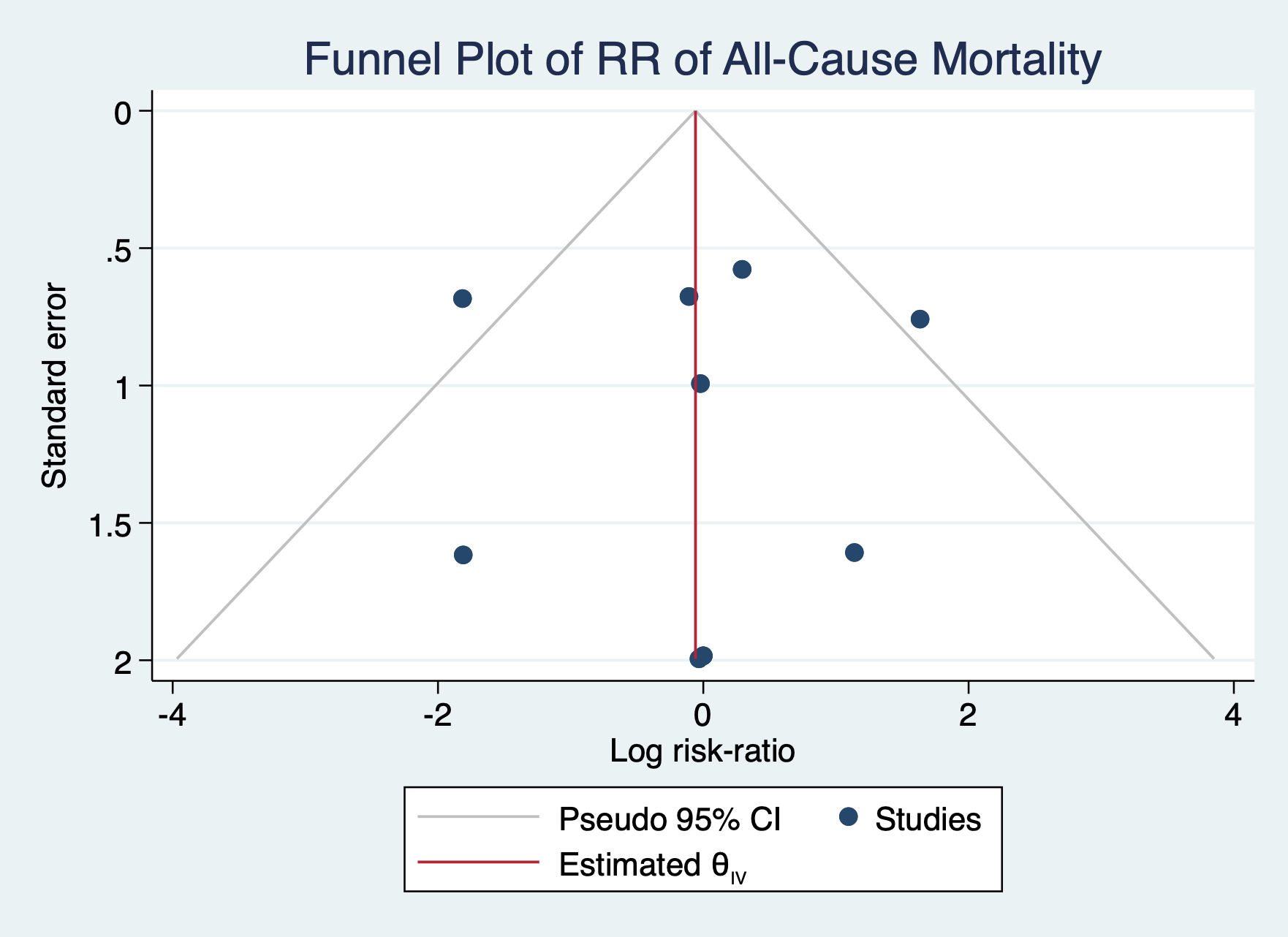**  **A** | **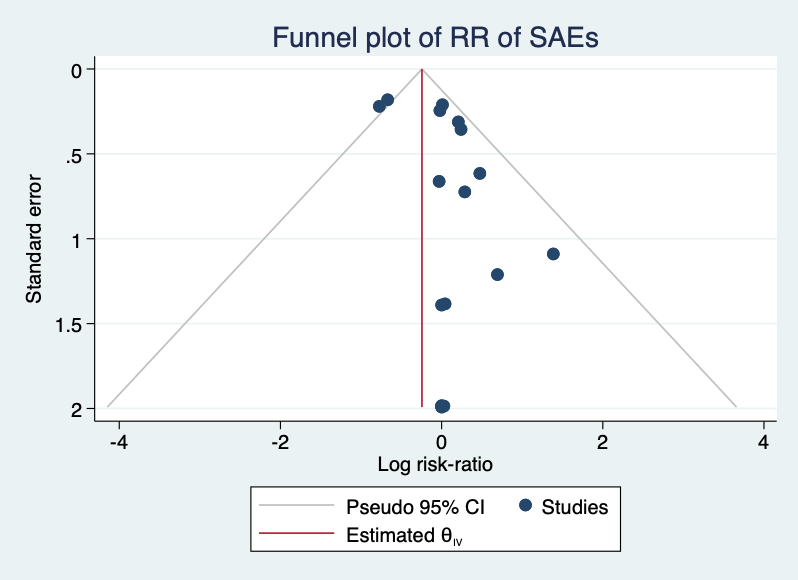**  **B** |
| --- | --- |
| 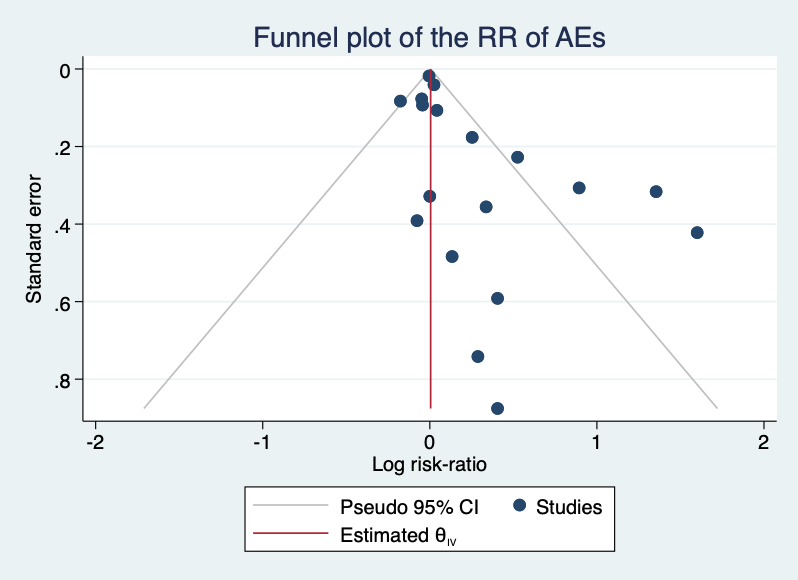  **C** | 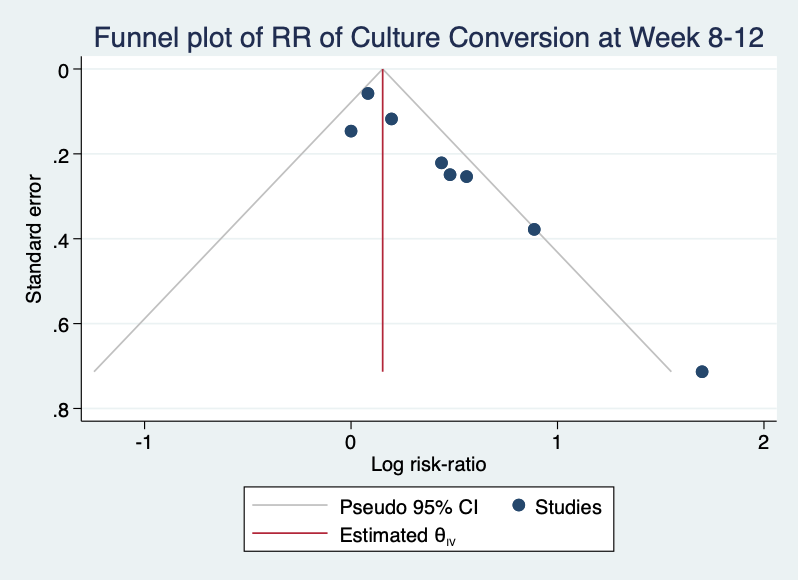  **D** |
| 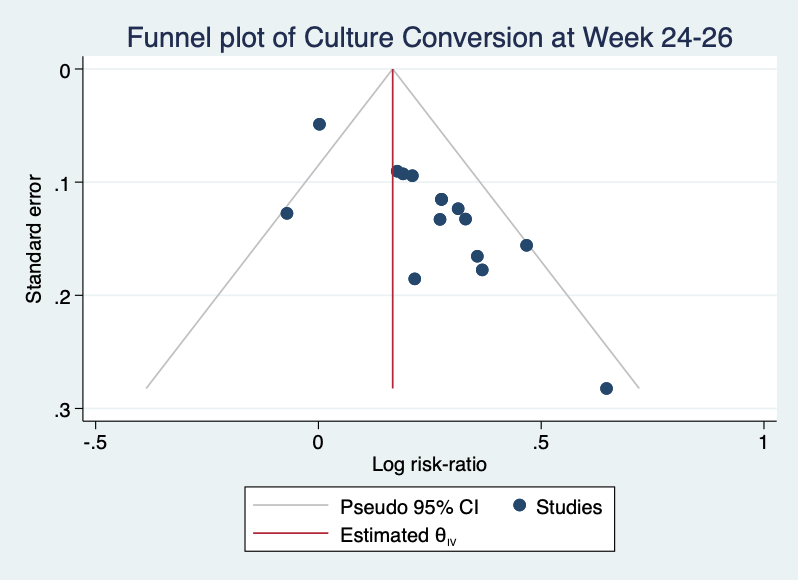  **E** | 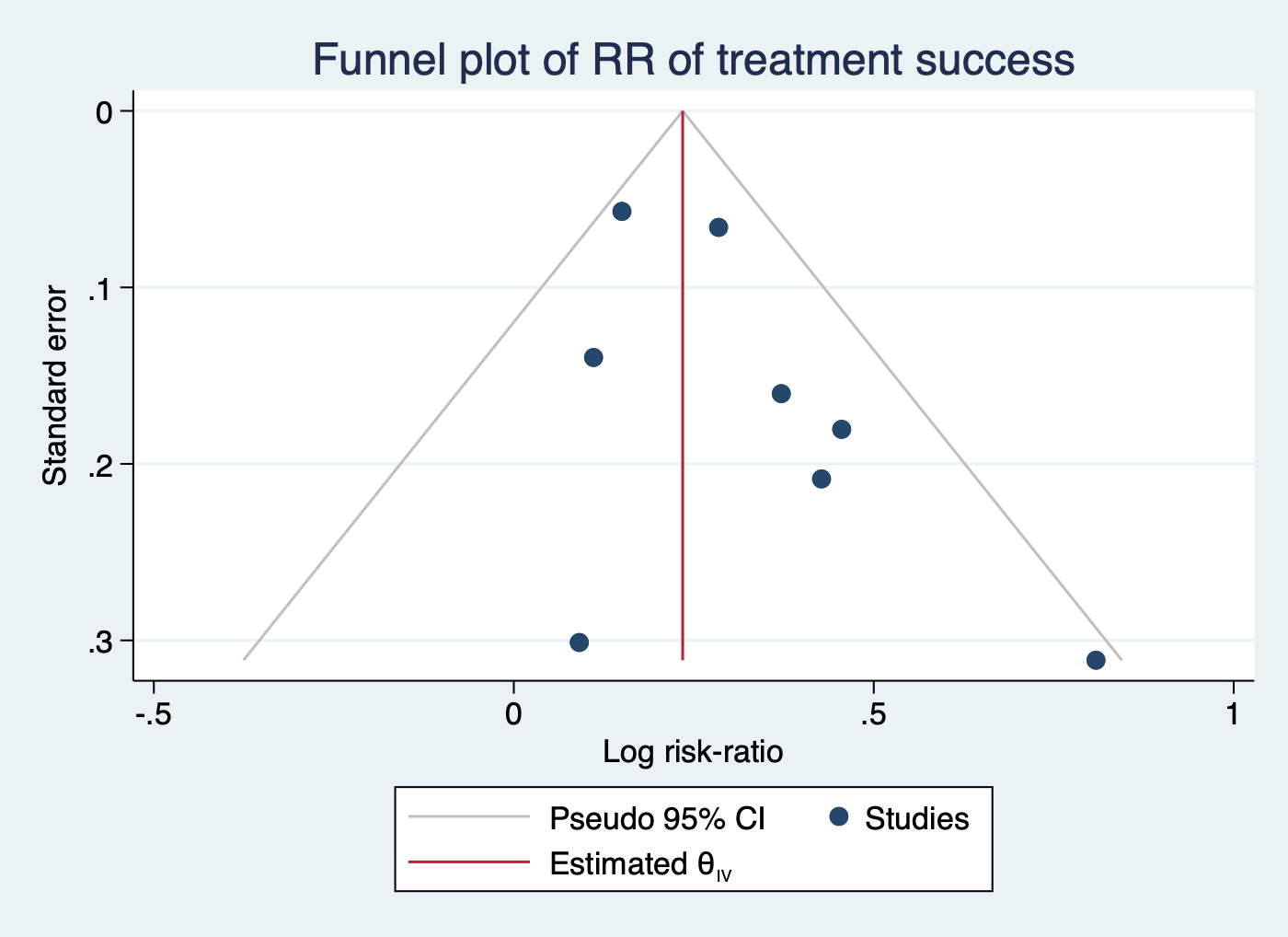  **F** |
| 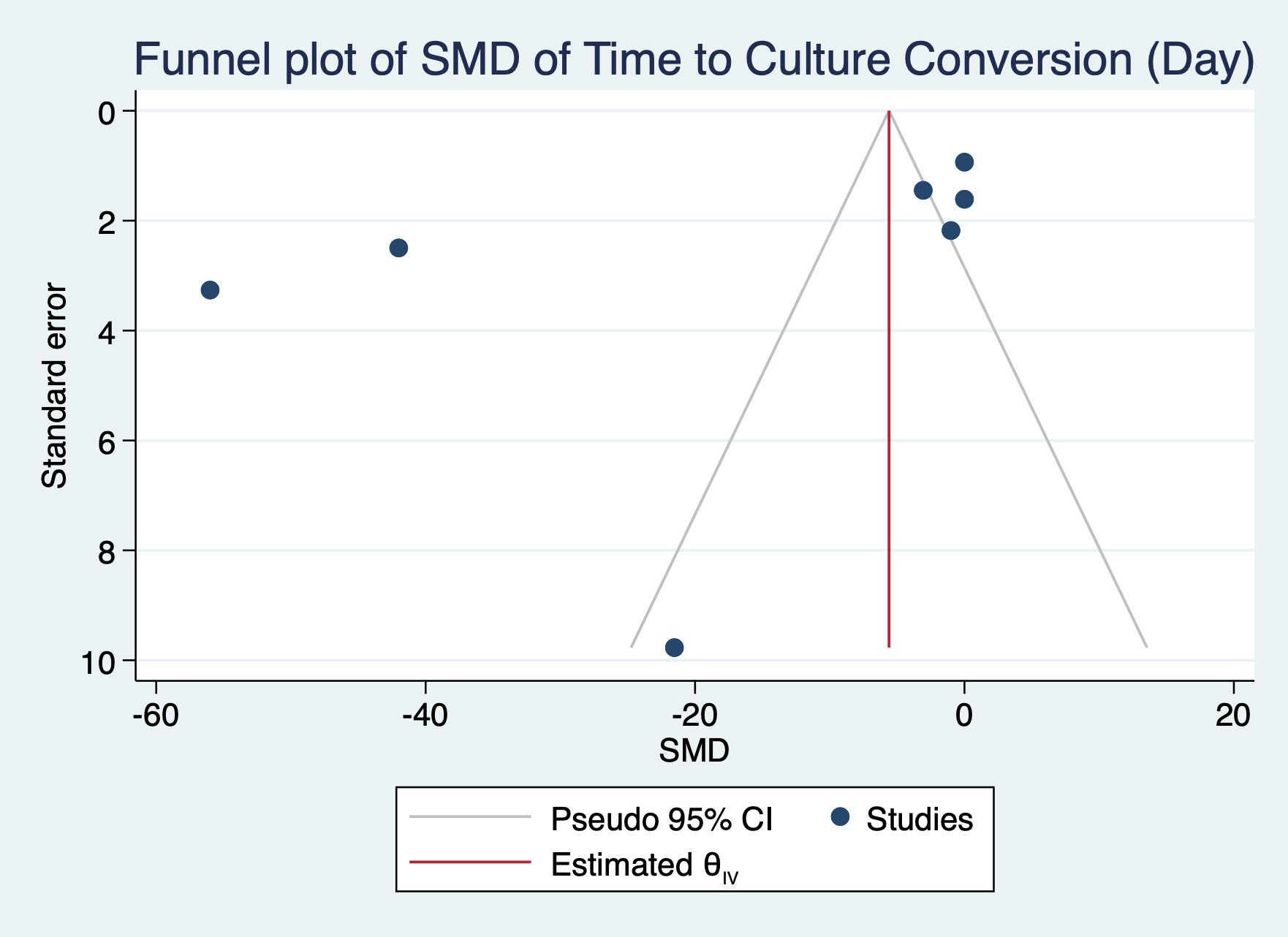  **G** |  |

**Fig. S2.** Funnel plots of safety and efficacy outcomes.

AEs: adverse events; RR: relative risk; SAEs: serious adverse events; SMD: standardized-mean difference.

Regression-based Egger test for small-study effects using an inverse-variance method for (A) all-cause mortality (*P* value = 0.9830), (B) SAEs (*P* value = 0.1562), (C) AEs (*P* value = <0.001), (D) sputum culture conversion at 8-12 weeks (*P* value = <0.001), (E) sputum culture conversion at 24-26 weeks (*P* value = <0.001), (F) treatment success (*P* value = 0.0723), and (G) time to culture conversion (*P* value = <0.001).
